# 

**Published:** 1896-03

**Authors:** 


SUBSCRIBE FOR THE

### International Dental Journal,

EDITED BY JAMES TRUMAN, D.D.S.

### Published by the International Dental Publication Company.

LOUIS JACK, PRESIDENT, Philadelphia.
BENJAMIN LORD, VICE-PRESIDENT, New York.
C. N. PEIRCE, TREASURER, Philadelphia.
GEO. S. ALLAN, SECRETARY, 51 West Thirty-seventh St., New York.

**Publication Office, 716 Filbert St., Philadelphia, Pa.**

STOCKHOLDERS.

| | | | |
|---|---|---|---|
| GEORGE EMORY ADAMS | South Orange, N.J. | W. K. LEECH | Philadelphia, Pa. |
| GEO. S. ALLAN | New York, N.Y. | *FRED. A. LEVY | Orange, N.J. |
| R. R. ANDREWS | Cambridge, Mass. | WILBUR F. LITCH | Philadelphia, Pa. |
| H. A. BAKER | Boston, Mass. | J. BOND LITTIG | New York, N.Y. |
| A. E. BALDWIN | Chicago, Ill. | BENJAMIN LORD | New York, N.Y. |
| W. C. BARRETT | Buffalo, N.Y. | GEO. W. LOVEJOY | Montreal, Ont. |
| *A. P. BEALE | Philadelphia, Pa. | JOHN S. MARSHALL | Chicago, Ill. |
| S. T. BEALE, JR. | Philadelphia, Pa. | H. J. MCKELLOPS | St. Louis, Mo. |
| C. S. BECK | Wilkesbarre, Pa. | CHARLES MCMANUS | Hartford, Conn. |
| G. V. BLACK | Jacksonville, Fla. | JAMES MCMANUS | Hartford, Conn. |
| C. F. W. BODECKER | New York, N.Y. | DAN'L NEAL MCQUILLAN | Philadelphia, Pa. |
| E. A. BOGUE | New York, N.Y. | FREDERIC A. MERRILL | Boston, Mass. |
| W. G. A. BONWILL | Philadelphia, Pa. | HORATIO C. MERIAM | Salem, Mass. |
| C. A. BRACKETT | Newport, R. I. | W. D. MILLER | Berlin, Germany. |
| THOMAS BRADLEY | New York, N.Y. | GEO. T. MOFFATT | Boston, Mass. |
| EDWARD C. BRIGGS | Boston, Mass. | A. L. NORTHROP | New York, N.Y. |
| ALBERT H. BROCKWAY | Brooklyn, N.Y. | W. E. PAGE | Boston, Mass. |
| A. E. BROWN | Chicago, Ill. | S. B. PALMER | Syracuse, N.Y. |
| WM. CARR | New York, N.Y. | C. B. PARKER | Brooklyn, N.Y. |
| GEO. H. CHANCE | Portland, Oregon. | D. D. PEABODY | Stoneham, Mass. |
| G. F. CHENEY | St. Johnsbury, Vt. | C. N. PEIRCE | Philadelphia, Pa. |
| DWIGHT M. CLAPP | Boston, Mass. | S. G. PERRY | New York, N.Y. |
| JOHN T. CODMAN | Boston, Mass. | W. A. PHREANER | Philadelphia, Pa. |
| CHAS. D. COOK | Brooklyn, N.Y. | CHAS. E. PIKE | Philadelphia, Pa. |
| J. N. CROUSE | Chicago, Ill. | W. E. PINKHAM | New York, N.Y. |
| EDW. T. DARBY | Philadelphia, Pa. | W. H. POTTER | Boston, Mass. |
| H. S. DRAPER | Boston, Mass. | CHAS. P. PRUYN | Chicago, Ill. |
| WM. H. DWINELLE | New York, N.Y. | H. C. REGISTER | Philadelphia, Pa. |
| A. R. EATON | Elizabeth, N.J. | F. A. ROY | New Orleans, La. |
| ALBERT T. EMERY | Boston, Mass. | Z. T. SAILER | New York, N.Y. |
| CHAS. J. ESSIG | Philadelphia, Pa. | L. D. SHEPARD | Boston, Mass. |
| J. N. FARRER | New York, N.Y. | GEO. R. SHIDLE | Pittsburg, Pa. |
| T. FILLEBROWN | Portland, Me. | EUGENE H. SMITH | Boston, Mass. |
| W. B. FINNEY | Baltimore, Md. | B. HOLLY SMITH | Baltimore, Md. |
| T. A. FLETCHER | New York, N.Y. | H. A. SMITH | Cincinnati, Ohio. |
| M. W. FOSTER | Baltimore, Md. | S. G. STEVENS | Boston, Mass. |
| CHAS. E. FRANCIS | New York, N.Y. | W. X. SUDDUTH | Minneapolis, Minn. |
| E. C. FUNDENBERG | Pittsburg, Pa. | EUGENE S. TALBOTT | Chicago, Ill. |
| W. F. FUNDENBERG | Pittsburg, Pa. | *AMBLER TEES | Philadelphia, Pa. |
| W. H. FUNDENBERG | Pittsburg, Pa. | J. D. THOMAS | Philadelphia, Pa. |
| E. S. GAYLORD | New Haven, Conn. | JAMES TRUMAN | Philadelphia, Pa. |
| J P. GERAN | Brooklyn, N.Y. | WM. H. TRUEMAN | Philadelphia, Pa. |
| A. H. GILSON | Boston, Mass. | W. W. WALKER | New York, N.Y. |
| S. H. GUILFORD | Philadelphia, Pa. | I. F. WARDWELL | Stanford, Conn. |
| JOHN I. HART | New York, N.Y. | G. W. WARREN | Philadelphia, Pa. |
| O. E. HILL | Brooklyn, N.Y. | T. S. WATERS | Baltimore, Md. |
| J. F. P. HODSON | New York, N.Y. | GEO. W. WELD | New York, N.Y. |
| J. MORGAN HOWE | New York, N.Y. | JULIUS G. W. WERNER | Boston, Mass. |
| ROBERT HUEY | Philadelphia, Pa. | JACOB L. WILLIAMS | Boston, Mass. |
| A. O. HUNT | Iowa City, Iowa. | *R. B. WINDER | Baltimore, Md. |
| LOUIS JACK | Philadelphia, Pa. | C. A. WOODWARD | New York, N.Y. |
| V. H. JACKSON | New York, N.Y. | J. A. WOODWARD | Philadelphia, Pa. |
| C. R. JEFFERIS | Wilmington, Del. | W. A. WOODWARD | New York, N.Y. |
| *C. A. KINGSBURY | Philadelphia, Pa. | W. J. YOUNGER | San Francisco, Cal. |
| W T. LA ROCHE | Harrington Park, N.J. | | |

***Deceased.**

The officers of the INTERNATIONAL DENTAL PUBLICATION COMPANY serve without salary, the investments of the stockholders merely guarantee the success of the enterprise, ALL PROFITS being devoted to the enlargement or improvement of the INTERNATIONAL DENTAL JOURNAL.

**The support of every dentist having the welfare of his profession at heart is earnestly solicited. Subscription, $2.50 per Annum.**

*THE INTERNATIONAL DENTAL PUBLICATION CO.*

